# Supplementary material for: The DACH1 Gene Transcriptional Activation and Protein Degradation Mediated by Transactivator Tas of Prototype Foamy Virus
Source: Viruses. 2023 Sep 8;15(9):1899. doi: 10.3390/v15091899 (PMC10534306; doi:10.3390/v15091899)
Supplement: Supplementary file 1 [file viruses-15-01899-s001.zip › viruses-2580086-supplementary.pdf]

Supplementary Materials

Table S1. The primers of DACH1 sequence deletion and mutation vector construction.

Vector	Sense primer (5′-3′)	Antisense primer (5′-3′)
pGL3-DACH1-dS1	TAGGGGTACCAAGGACTTTCCAAAACTACAGC	
pGL3-DACH1-dS2	TAGGGGTACCCATCCACATCAAGGCAAGC	GCGAAGATCTCACAAAAGTGTCCCGCAAG
pGL3-DACH1-dS3	TAGGGGTACCCCACTTGAAAACTGTCCTG	
pGL3-DACH1-dS4		GCGAAGATCTCTGGACGAGTTGTTGTTGT
pGL3-DACH1-dS5		GCGAAGATCTAGAGGGAGCGTGCGAGG
pGL3-DACH1-dS6	TAGGGGTACCAAGGACTTTCCAAAACTACAGC	GCGAAGATCTCTTCTCTTCTACTACTACCGTTA
pGL3-DACH1-dS7		GCGAAGATCTCTAAACAATCGCCCGCC
pGL3-DACH1-dS8		GCGAAGATCTGTAAGCAAAGCGTAATGTTC
pGL3-DACH1-mS1	CGAGTTCatacgtaGATAATTTGGGTTAATACTAGTGAGT	AATTATCtacgtatGAACTCGGAGCAGAGACTCCG
pGL3-DACH1-mS2	CGGAGTCgagtagaCGAGTTCCTGGTTGGATAATT	GAACTCGtctactcGACTCCGAGAGCGCGAGA
pGL3-DACH1-mS3	TCTCTGCTCatctggaCTGGTTGGATAATTTGG	TCCAACCAGtccagatGAGCAGAGACTCCGAGAGC
pGL3-DACH1-mS4	TTCCTGGTTGtcgccggTGGGTTAATACTAGTGAGTGAGGT	TTAACCCAccggcgaCAACCAGGAACTCGGAGC
pGL3-DACH1-mS5	GATAATTgtttggcATACTAGTGAGTGAGGTTTTTGC	ACTAGTATGCCAAACAATTATCCAACCAGGAACTCGG

Note：Lowercases in primer sequence represent mutation sites.

Table S2. The primers for DACH1 shRNA lentivirus vectors

Vector	Sense primer (5′-3′)	Antisense primer (5′-3′)
DACH1-shRNA01	GATCCGCCTCCTAAGAGGACTCAACTTCCTGTCAGATTG	AATTCAAAAAGCCTCCTAAGAGGACTCAATCTGACAGGAAGTTG
DACH1-shRNA02	GATCCGCACTTGAGTTTGAGACGACTTCCTGTCAGA	AATTCAAAAAGCACTTGAGTTTGAGACGATCTGACAGGAAGTCGT
DACH1-shRNA03	GATCCCTGTTGAAAGTTGCCATAGCTTCCTGTCAGACTA	AATTCAAAAACTGTTGAAAGTTGCCATAGTCTGACAGGAAGCTAT

Table S3. The primers for DACH1 SUMOylation site-directed mutation

Primer	Sequence (5′-3′)
DACH1-mSF1	AAGGTGAAAAAAATCagaTTAGAAGCCAT
DACH1-mSR3	AGTTGCTCATGGCTTCTAAtctGATT
DACH1-mSF1	CTGGAAAAAACTGAGCTGaggATGGATTT
DACH1-mSR1	TCTTTCCCTTAAAAAATCCATcctCAGCTC
DACH1-mSF1	TAGTTCAAAAGAGGCTAaggAAGGAG
DACH1-mSR2	TGCCTTCTTCTCCTTcctTAGCCT

Note: Lowercases in the sequence represent the lysine to be mutated.

-2000 CATATTTCTATTTTTCGGTAGACAGTTCCTGCACTATTTGACACTGCGATTCACAGAGTTCAAAGGCTTAAAATGATATAGAGAAATGTG

-1910 ATCATTTCAACACCAGAATGGCAAAAATTTTATTATAAAATTTCCCAACCCTGTGAACCTGCTACTAACAGTTTATTTTAAAAATCTGTC

-1820 AAATGCTAAAGCATTGCTTCATATCTTATGAACTTACTGAACATGTGTGGACCGGTTCAAAGGCTAACTAATTGAAGATACTTGGAGAC

-1731 ATGAGCAGCAAATAGATTTGACCCATATTCAAATGATA**TGTTAGTCA**AAAATCCTTGAATAAATACTTCAGTGCAACAAGTTAGAAAA

AP-1 binding site

-1648 GAAAAGAAAAGGAAAGAGGAAGGAAAGGAGGA**ATAGAGTCA**GTCTAAACTCAGCCTCATGTTGTTTTAAGCACAGCTACCCAAGT

AP-1 binding site

-1558 CTT**TACAAGTCA**ACTAGTGAGCTTTGTTTTTGTTCTTCCCATTATTTACAAAACATATATTTACTGACAAATTGAAAAGGACTTTCCAAA

AP-1 binding site

-1468 ACTACAGCAGCACTTTTCAAACATGAAAAAAAAAAAAAAAAAAAACTATTGTGTTGTCCAAAAAACACATTAGCAATTGGCTTTGGGTC

-1381 TTAAAGGACATGAGGATAGTTGGTTACTGTGTAAGACAGGTGAATTAATTATAGAGTTTTGGGTAAGAAAACCCTTTTTCCACTTTCTA

-1648 AATCTTCAAGGATACCTTATCTCCTTGATAGCTCTAGGTTTAACAGGTATTTC**TGACTGGCTTTTGG**GGAGACAGTGAAGTTATTCTAA

CAAT box

-1292 ATCATCCACATCAAGGCAAGCATTTCTTTCCACAACCTATTAGGAAATATGGAATTGAATACAGAGCTTTCCATTATTCTTAAGCGATTT

-1113 CGGAC**TGACTTTCT**TCTCACCACTTGAAAACTGTCCTGGAATTTTCACTATC**TTTATATAAA**TAAATAATTTAGTAGAGAAATAAACAA

AP-1 binding site **TATA box**

-1024 TCAGATGCAAAAGAACATTACGCTTTGCTTACATGTGAATTTTCCTCAAAATAATTTCTGAATAATCGTGAAGTGGGGGCTGTGTTTTCG

-934 TTTCTAGCTCATTGCCAAAACGCGTGGAGATGGATCCTAAATCCTGGAAGGAGAGAGCTCGGCGCGCTCAGGCTCCTCTTTAAGTTGG

similar with Kip2-TRE

-846 CGGGCGATTGTTTAGAAGCTTTGGAAAAAATTACCAGTCCCC**GCGGACGAAAT**GGAATGTGAGACATGCAATCTAACGGTAGTAGTA

similar with IP-TRE

-759 GAAGAGAAGTAGATGA**TCTCCCTCTCCTT**T**TCCGATCGC**CACTTCTGTGTCCCCGGCTTTTCCTCTCCGTCCCTCCCACAGTTTCTTTC

NF-κB binding site **similar with Kip2-TRE**

-670 CGTATCCTCCCCCTCCCCTCCTCCTTTCCTCGCACGCTCCCTCTCCAGTGTCTCGCGCTCTCGGAGTCTCTGCTCCGAGTTC**CTGGTTG**G

similar with IP-TRE

-580 ATAATTTGGGTTAATACTAGTGAGTGAGGTTTTTGCGGCTTCAAAGGGTATTTCAAGTTTCCGGAGCTCCTCCCCTCTGCACCGTGTGAC

-490 AACAACAACTCGTCCAGCCGGCAGCCTGCACTTTTCAGCTCATTTTCTCTCTGTCATTCCCCTCTCAATCTTTGATCAATGTACTTGCCA

-400 GGGAGAGCCCAAGTCCTTCAAACCTCCTCCTTTTCACCTTCATCCTTAACTTTGTGCTAGAGCGAGACCCACACAACAACAGCCGACC

-312 C**TCCCCGCCCC**ACCCCCACCCCCAAACCAGCCCTCGATCCCAGCCCCCGGAGAGGACTCGCATTTCGACTTGCGGGACACTTTTGTG

GC box

-225 CGTTCCTCTCCAGAGCGCCTCTCGTGCTCGCCCCTCTTGCGCTCGCTCTTTATTACCTTCACCTCCTTTTCTCCCCCTTCTCTCCCTTTCTC

-133 CTTCTCGTTCTCTCCCGGAGTTGTTGTTGCCCCCCTCGCTCCTTCTCCCCCCTTTTTTCCCCTTCCCCTCCCGGGGGTGTGTGGCAACTTT

-42 TCCTCTCGCTTCTCCTCCGTCTGTTTCCCCTTATATGTGACCATGGCAGTGCCGGCGGCTTTGATCCCTCCGACCCAGCTGGTCCCCCCT

translation initiation site

+49 CAACCCCCAATCTCCACGTCTGCTTCCTCCTCTGGCACCACCACCTCCACCTCTTCGGCGACTTCGTCTCCGGCTCCTTCCATCGGACC

+138 CCCGGCGTCCTCTGGGCCAACTCTGTTCCGCCCGGAGCCCATCGCTTCGGCGGCGGCGGCGGC

Figure S1. Promoter sequences of *DACH1*. The underlines in sequences are regulatory elements or compared with Kip2-TRE or IP-TRE.

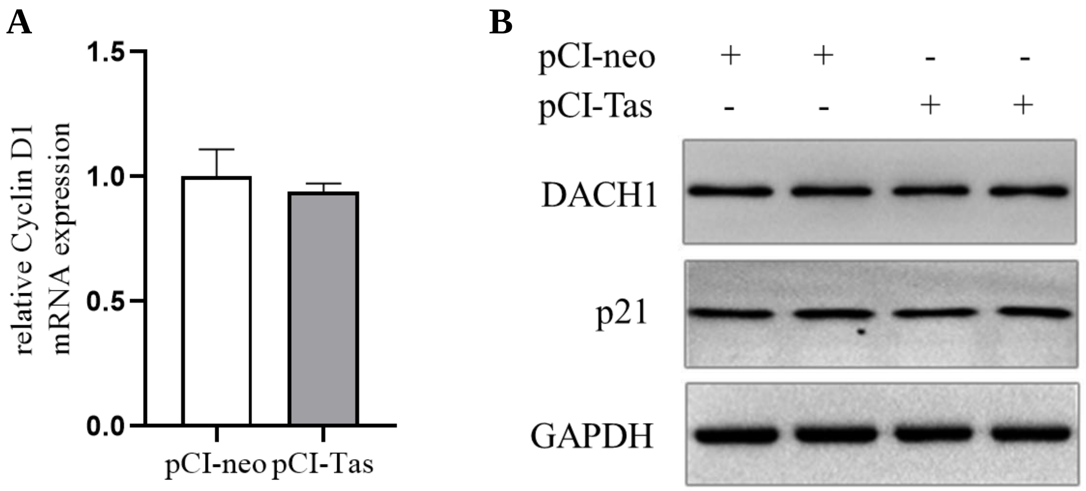

Figure S2. The mRNA expression of Cyclin D1 and protein expression of p21 after pCI-Tas transfection. (A)The mRNA expression of Cyclin D1 was unchanged after Tas transfection. HeLa cells were plated in a 12-well plate at 2×10[5], pCI-neo (1 μg) or pCI-Tas (1 μg) was transfected into the cells, and RT-qPCR was utilized to detect the mRNA expression of Cyclin D1. (B) Tas transfection had no influence on the protein expression of p21. HeLa cells were plated in a 6-well plate at 5×10[5], and transfected with pCI-neo (3 μg) or pCI-Tas (3 μg). After 48 h of transfection, the protein of cells was extracted and western blot was performed to assess the protein expression of p21, DACH1, and GAPDH.
